# State-of-the-art imaging for diagnosis of metastatic bone disease

**DOI:** 10.1007/s00117-020-00666-6

**Published:** 2020-03-24

**Authors:** Amanda Isaac, Danoob Dalili, Daniel Dalili, Marc-André Weber

**Affiliations:** 1 Department of Clinical Radiology, Guy’s & St Thomas’ Hospitals, London, UK; 2 School of Biomedical Engineering & Imaging Sciences, Kings College London, London, UK; 3 London Deanery, London, UK; 4 Institute of Diagnostic and Interventional Radiology, Paediatric Radiology and Neuroradiology, University Medical Centre Rostock, Rostock, Germany

**Keywords:** Metastasis, Skeletal, Imaging, Bone, Health-care costs, Whole-body, Metastasierung, Skeletal, Bildgebung, Knochen, Kosten der medizinischen Versorgung, Ganzkörper

## Abstract

Metastatic bone disease (MBD) is common—it is detected in up to 65–75% of patients with breast or prostate cancer, in over 35% of patients with lung cancer; and almost all patients with symptomatic multiple myeloma have focal lesions or a diffuse bone marrow infiltration. Metastatic bone disease can cause a variety of symptoms and is often associated with a poorer prognosis, with high social and health-care costs. Population-based cohort studies confirm significantly increased health-care utilization costs in patients presenting with cancer with MBD compared with those without MBD. The prolonged survival of patients with bone metastasis thanks to advances in therapy presents an opportunity for better treatments for this patient cohort. Early and accurate diagnosis of bone metastases is therefore crucial. The patterns and presentation of MBD are quite heterogeneous and necessitate good knowledge of the possibilities and limitations of each imaging modality. Here, we review the state-of-the-art imaging techniques, assess the need for evidence-based and cost-effective patient care pathways, and advocate multidisciplinary management based on collaborations between orthopedic surgeons, pathologists, oncologists, radiotherapists, and radiologists aimed at improving patient outcomes. Radiologists play a key role in this multidisciplinary approach to decision-making through correlating the tumor entity, the tumor biology, the impact on the surrounding tissues and progression, as well as the overall condition of the patient. This approach helps to choose the best patient-tailored imaging plan advocating a “choose wisely” strategy throughout the initial diagnosis, minimally invasive treatment procedures, as well as follow-up care plans.

## Abstract (de)

Eine Knochenmetastasierung ist häufig – bei bis zu 65–75 % der Patienten mit Brust- oder Prostatakrebs, bei über 35 % der Patienten mit Lungenkrebs; und fast alle Patienten mit symptomatischem multiplem Myelom weisen fokale Läsionen oder eine diffuse Knochenmarkinfiltration auf. Knochenmetastasierung kann eine Vielzahl von Symptomen hervorrufen und ist häufig mit einer schlechteren Prognose sowie hohen sozialen und medizinischen Kosten verbunden. Bevölkerungsbasierte Kohortenstudien bestätigen einen signifikanten Anstieg der Kosten für die Inanspruchnahme der Gesundheitsversorgung bei Patienten mit Knochenmetastasierung im Vergleich zu Patienten ohne ossäre Metastasen. Ein längeres Überleben von Patienten mit Knochenmetastasen aufgrund von Fortschritten in der Therapie bietet die Möglichkeit, diese Patienten besser zu behandeln. Eine frühzeitige und genaue Diagnose von Knochenmetastasen ist daher von entscheidender Bedeutung. Muster und Manifestation der Knochenmetastasierung sind sehr heterogen und erfordern eine genaue Kenntnis der Möglichkeiten und Grenzen der einzelnen Bildgebungsmodalitäten. In der vorliegenden Übersicht werden die neuesten bildgebenden Verfahren vorgestellt, die Notwendigkeit evidenzbasierter und kosteneffektiver Behandlungswege erörtert und ein multidisziplinäres Management propagiert. Dabei arbeiten Orthopäden, Pathologen, Onkologen, Strahlentherapeuten und Radiologen zusammen, um die Ergebnisse für die Patienten zu verbessern. Radiologen spielen eine Schlüsselrolle in diesem multidisziplinären Ansatz zur Entscheidungsfindung, indem sie die Tumorentität, die Tumorbiologie, die Auswirkungen auf das umgebende Gewebe und das Fortschreiten der Erkrankung sowie den Gesamtzustand des Patienten miteinander in Beziehung setzen, um die beste auf den Patienten zugeschnittene Bildgebungsstrategie zu wählen. Das bedeutet „image wisely“ sowohl bei der Erstdiagnose als auch bei minimalinvasiven therapeutischen Eingriffen und im Rahmen von Nachsorgeplänen.

## Background

Bone metastases are common, can cause a variety of symptoms, and are often associated with a poorer prognosis. Nearly all patients who die of prostate cancer have metastatic bone disease (MBD; [1]). An up to tenfold surge in the risk of death depending on the histology of the primary malignancy [2] has been reported, with a documented negative impact on progression-free survival [3]. Studies stipulate that the presence of MBD increases the cost of patient care by 62–300%, with an average of $ 11,820 higher annual health-care costs than for controls [4–7]. The estimated lifetime cost of a skeletal-related event (SRE) was reported to be up to 12,000 $ per patient with lung cancer in 2004. A more recent study shows that on average, the total 6‑month cost of treating patients with SREs was $ 43,746 compared with $ 25,956 in the matched control cohort [8]. Over 1.9 billion dollars was spent on managing MBD in prostate cancer patients in the United States in 2004 [6]. Such costs have increased over time owing to the prolonged survival and development of more innovative treatments that can now be offered to the patient when compared with previous decades. According to a recent study by Kraywinkel et al., for most cancer sites and age groups there are significant positive trends in survival. Age-standardized survival for all cancers combined increased by 7.1% units for women and 10.8% units for men [9]. The 5‑year survival rates reach 20% with multiple bone metastases and 40% when presenting with a solitary lesion [10]. Variations exist depending on the original type and grade of malignancy. The prevalence of patients with MBD presenting to health-care facilities has increased as a result, which has an impact on overall workload and demands on services. There are notable cost variations between countries depending on locally agreed prices, treatment pathways, and reimbursement structures, which add to the complexity of these health economics comparisons [11].

The majority of patients with MBD present with either pain or cord compression [12–15], when fractures occur (pathological fracture), when routine staging scans are performed (for newly diagnosed malignancies elsewhere in the body), or when restaging interval scans are performed (to assess the response to various therapies). The SREs related to MBD comprise pathological fractures in 4–7% of patients [3], spinal cord or nerve root compression, hypercalcemia, and anemia (due to bone marrow suppression), as well as the need for radiotherapy, orchiectomy, or more invasive therapies. Radiotherapy was by far the most common SRE, received by 85% of all MBD patients, with nearly 50% of these patients receiving radiotherapy within 2.5 years of their MBD diagnosis [4]. Persistent pain resulting from bone metastases is often severe, progressive, and multifocal [16], requiring multidisciplinary therapy and repeated treatments.

Treatment options may be curative (surgery, chemo-, radiotherapy), locally curative (radiotherapy, ablation), or palliative (radiotherapy, chemotherapy, arterial embolization, stabilization with forms of cementoplasty such as kyphoplasty, and/or vertebroplasty, which can be combined with radiofrequency ablation, cryotherapy, internal fixation, or systemic drug therapy including nonsteroidal anti-inflammatory drugs, opioid analgesics, and adjuvant drugs). It is therefore important to develop agreed upon local pathways for referral of these patients and to communicate this information widely across their local clinical settings. This facilitates discussions between various disciplines and expedites a patient-specific tailored management plan, with potential cost and time savings. Advances in imaging have increased the sensitivity and specificity of detecting bone lesions, increasing the positive predicative value of imaging. With these advances, expectations of patients and carers with regard to medical care and health-care professionals have also increased.

### Aim of the study

The purpose of this article is to help radiologists and clinicians utilize state-of-the-art imaging to determine whether a bone lesion seen on imaging is a bone metastasis (from a confirmed/unknown primary), to guide further management, and to initiate appropriate referral to relevant multidisciplinary teams. We discuss the benefits of each imaging modality, describe new and evolving image-guided therapies, as well as highlight the importance of considering the cost effectiveness of diagnostic and interventional modalities currently available to ensure we can offer patients a sustainable, effective, and patient-tailored service.

We advocate developing local pathways for prompt discussion of the imaging and clinical findings, and immediate clinical management of these patients where necessary.

## Terminology

The phrase “metastatic bone disease (MBD)” is used throughout this article to refer to focal bone lesions (a) identified on imaging in patients with a known primary malignancy and/or (b) confirmed histologically following a biopsy.

The term “skeletal-related event” (SRE) refers to metastasis of a tumor to bone and/or its clinical effects, for example, when the cancer has spread to the bone (metastasis) to weaken it, cause pain, and increase fracture risk.

## Incidence and prevalence of MBD in Europe

**Fig. 1:**
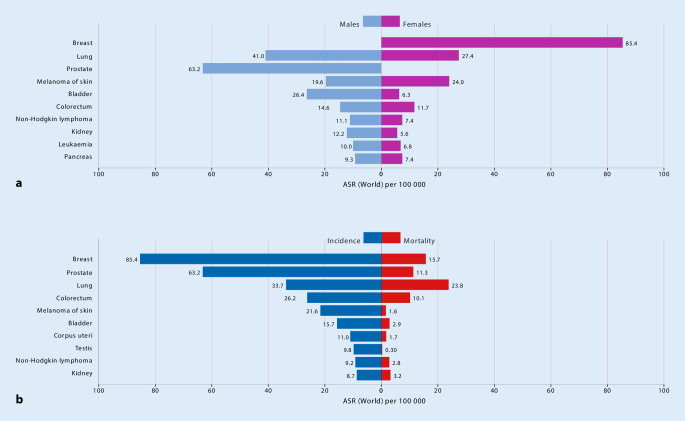
**a** Age-standardized (world) incidence rates per sex, top 10 cancers. **b** Age-standardized (world) incidence and mortality rates, top 10 cancers. (The Global Cancer Observatory, Cancer in Germany register 2018–2019; https://gco.iarc.fr/today/data/factsheets/populations/276-germany-fact-sheets.pdf. This content is not part of the Open Access License.)

Metastatic bone disease remains the commonest bone lesion in adults, with presentations to almost every hospital in Europe. Primary malignant bone tumors remain rare in all European countries ([17–19]; Table 1; Fig. 1). Therapy such as bisphosphonates have significantly changed the natural history of bone metastases by reducing SREs, so that the majority of patients now live with bone metastases for several years. The 5‑year survival rates have been reported to be 20% with multiple bone metastases and 40% in cases of a solitary lesion [20].

**Table 1:** Epidemiological indicators: total number of cancer cases in Germany in 2013 (modified according to [21])

*New cases*	*Women*	*Men*	*Death*	*Women*	*Men*
Absolute number	229,920	252,550	Absolute number	101,779	121,314
Mean age at diagnosis	67.2	68.3	Mean age at diagnosis	74.3	73.3
Raw rate	558.4	639.9	Raw rate	247.2	307.7
Age-standardized rate^a^	351.2	434.1	Age-standardized rate^a^	126.7	196.5
Current trend	+0.8%	−0.5%	Current trend	−0.7%	−1.2%
Age-standardized rate^a^	327.9	447.6	Age-standardized rate^a^	126.8	211.8
Prognosis for 2020 (absolute number)	244,100	274,900	Prognosis for 2020 (absolute number)	−0.8%	−1.5%
*Survival*			*Prevalence*		
Relative 5‑year survival	66%	61%	Relative 5‑year prevalence	791,770	803,780
Relative 10-year survival	61%	57%	Relative 10-year prevalence	1,334,320	1,334,270

Total for cancer excluding nonmelanotic skin cancer (Germany, 2013); comparison of selected results for the European Union, 2012 (incidence) and 2013 (mortality)

^a^Average annual change in age-standardized rate between 2003 and 2013

The commonest sites of MBD are the spine and pelvis. The prevalence of MBD depends on the primary malignancy—the commonest originating from breast, prostate, and lungs (68% of cases documented in the United States in 2017 and in Germany in 2018 [22]; https://gco.iarc.fr/today/data/factsheets/populations/276-germany-fact-sheets.pdf).

Bone metastases occur in 50% of patients with cancer, and among these, 40–70% are vertebral lesions [23], with unknown primary in 10% of patients [24]. In adults the primary tumors causing vertebral involvement are in the breast (22%), lung (15%), prostate (10%), lymphatic system (10%), connective tissue (9%), kidney (7%), and gastrointestinal tract (5%). Most of the metastases are located in the thoracic spine, less frequently in the lumbar, and rarely in the cervical spine (factors 4:2:1; [25]). Metastatic bone disease can occur at the time of presentation (metasynchronous) or years after treatment of the primary malignancy—primarily encountered with breast metastasis (metachronous). The MBD lesions are classified into lytic (lucent) or sclerotic (dense) metastasis, although features often coincide. This depends on the balance between osteoclastic activity (causing bone resorption), osteoblastic activity (causing bone deposition), as well as reactive bony changes (necrosis, fibrosis, or response to therapies; [26]). Early and accurate diagnosis of bone metastases is therefore crucial; however, the pattern is very heterogeneous and necessitates good knowledge of the possibilities and limitations of each imaging modality. Furthermore, reliable imaging parameters to predict therapy response in cases of bone metastases have not yet been elucidated in large randomized controlled clinical trials.

## History and clinical features

Patients with MBD and SREs may present to hospitals for various reasons. These patients can also present through various clinical settings including accident and emergency, orthopedic clinics, general practitioners, and community physicians. Metastatic bone disease occurs via hematogenous (arterial or venous) spread and less likely by direct extension (e.g., breast metastasis to ribs, soft tissue sarcomas to surrounding bones, gynecological malignancy spread to the bony pelvis). Many of these lesions remain asymptomatic for years. Some patients may present with gradual or acute bone pain or focal swelling and/or focal deformity. Pain could also be a mode of presentation when there is a pathological fracture in the affected bone. Sometimes, patients come to medical attention following trauma to the area and associated pain. The trauma itself is probably incidental in these cases. Some lesions are incidental findings on imaging examinations performed for other clinical reasons. The majority of skeletal metastases are detected during staging scans with computed tomography (CT), positron emission tomography combined with computed tomography (PET/CT), or during interval scans performed to assess the response to treatment. For this reason, it is important for reporting radiologists to critically evaluate the bones on all imaging examinations performed regardless of the clinical indication. Other presentations of skeletal metastases include hypercalcemia, spinal cord compression, and/or cauda equina syndrome.

Important considerations in the history include the growth rate of the tumor, the underlying oncological history, the estimated survival times, and the eligibility for surgery (should pathological fractures occur). A standardized checklist completed primarily by the treating oncology team and discussed with the radiologists and surgeons is worth compiling when feasible.

Imaging plays a crucial role at different stages of the disease. The purpose of imaging of MBD includes:

- To detect the presence of MBD at the earliest time point possible.
- To assess any possible differential diagnosis.
- To assess the lesion(s): local spread—cortical breach—impact on performance and function—impact on the surrounding structures (particularly the spine, neurovascular bundles, and other focal anatomical features that may influence the decision for surgery).
- To quantify the extent of disease load (mono-, oligo- or poly-ostotic).
- To assess the risk of a pending fracture or confirm the presence of a fracture and to help with planning further management.
- To plan the biopsy site (if required): compartment—areas to biopsy/target—friability—lucent or sclerotic—which biopsy tools to use.
- To determine the appropriateness of surgery and weigh up the various surgical and conservative treatment options. Skeletal metastases differ from primary malignant bone lesions in their surgical management. The majority of surgery for oligo- or poly-metastasis MBD aims to stabilize the bone affected by metastasis rather than to excise the lesion.
- To assess treatment monitoring through interval imaging.

## Primary imaging and the current imaging repertoire

Early imaging and diagnosis can reduce morbidity and/or mortality related to MBD [27]. Imaging strategies therefore aim at early and accurate detection, quantifying the extent of disease load, identifying the primary lesion, and assessing response to treatments. In terms of financial impact, these new innovations and modalities for imaging are likely to be cost effective as their influence on patient-specific targeted therapy is gradually being validated and endorsed, and their availability is increasing.

### Conventional plain radiographs: tumor detection and evaluation

Radiography is the principal imaging modality and can help diagnose MBD by providing information about the location, bone response (lytic or sclerotic), size, and number of lesions, as well as any possible pathological fractures and/or soft tissue involvement. In a significant number of cases, additional imaging is necessary. A bone lesion in a patient with any known primary malignancy should be considered a bone metastasis unless atypical features are present. Often, MBD itself is not visible on radiographs unless the tumor is mineralized or leads to a clear osteolysis as in myeloma [28] focal lesions, which is depicted when more than 50% of the bone substance has vanished [29]. The detection and diagnosis of the tumor are therefore dependent on the effect of the tumor on the host bone [30]. The extent of bone destruction caused by the MBD lesion rather than the tumor itself is what is often depicted on radiographs. For purely medullary tumors, there must be a destruction of at least 50% of the trabecular bone architecture before a tumor becomes visible on radiographs [31]. Small lesions are easily missed on radiographs, particularly in patients with low bone mineral density (osteoporosis). The trabecular bone density being higher in the epiphysis and metaphysis when compared with the diaphysis renders tumors in the epiphysis and metaphysis easier to detect than those in the diaphyseal medulla, owing to better contrast of the lesion against the adjacent normal trabeculae [30]. Likewise, bone tumor diagnosis on radiography is also delayed when the lesion is located in flat bones, the axial skeleton and ribs, because the host bone changes are difficult to appreciate with superimposition of the surrounding structures in such anatomical areas with relatively more complex anatomy [32].

### Cross-sectional imaging: MRI and CT

Magnetic resonance imaging (MRI) and CT can also be used to demonstrate the presence or absence of cortical destruction and/or a periosteal reaction. This would help to differentiate benign from malignant tumors when these features are difficult to determine on radiographs. Most often, CT is performed as part of the routine staging protocol during the initial work-up of any cancer diagnosis, and thus it is commonly the first imaging modality to detect the bone lesion suspected of representing MBD. Staging imaging examinations in the case of malignant bone tumors are usually performed after obtaining a histological diagnosis or when investigating malignancy of unknown origin [24]. Magnetic resonance imaging has superior sensitivity and specificity for tissue characterization; however, when MRI is not available or contraindicated, CT can provide similar information about the extent of the tumor. Computed tomography is particularly useful for further characterization of tumors with mineralized matrix and of sclerotic tumors. Most recently the application of dual energy CT virtual non-calcium algorithms has proven to be a valuable tool in the assessment of MBD lesions and disease load [33–35] as well as in identifying focal biopsy targets [36].

Moreover, CT is also useful for further characterization of tumors in the cortex, paracortical, and periosteal locations. It is often necessary to use CT for the characterization of tumors in the ribs, posterior elements of the spine, and other flat bones with a higher cortex/medullary bone ratio. In the case of some bones like the ribs and phalanges, CT may perform better than MRI, because of higher spatial resolution and fewer motion artifacts [37]. Owing to the higher resolution, CT also may perform better than MRI in the small bones of the hands, feet, or even in the skull. Computed tomography is particularly useful in the assessment of spine metastasis thanks to its sensitivity in cortico-medullary differentiation, as well as the ability to detect fractures [38]. Assessing stability is crucial for planning of further therapies (surgery, vertebroplasty, chemotherapy, or radiotherapy). There is further evidence that surgical decompression for spinal cord compression from metastatic disease before radiation therapy results in improved neurological outcomes and improved wound healing, with fewer complications. However, CT still fails to adequately assess the spinal cord if there is any concern for myelomalacia or impingement, and MRI should therefore be considered for patients presenting with new focal or widespread neural compromise.

In the case of metastatic bone disease, MRI is currently the best imaging modality to depict diffuse bone marrow involvement. Innovations in MRI such as whole-body diffusion-weighted imaging (DWI) and chemical-shift imaging have had a tremendous impact on cancer detection and management and are being integrated into mainstream routine imaging [27]. The degree of edema on MRI is not in itself a measure of the malignant potential of a bone tumor as this may be due to secondary infection of the lesions, to a pathological fracture, or to concomitant osteoarthritis [27, 37].

**Fig. 2:**
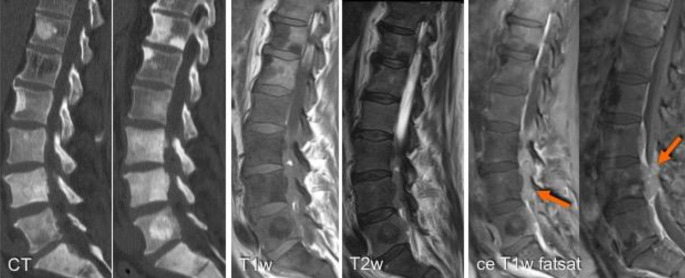
Computed tomography (*CT*) and multiparametric magnetic resonance imaging including T1-, T2- as well as pre- and postcontrast T1-weighted (*T1w*) fat-saturated (*fatsat*) imaging. Note the variable appearances of the lesions on T1, variable enhancement patterns, and superior demonstration of the soft tissue component of the metastatic bone disease at L3 and L4, extending into the spinal canal and posterior elements (*arrows*)

Magnetic resonance imaging is the best imaging modality for assessment of locoregional disease, as it allows for accurate assessment of the extent of the disease and the effect of the tumor on the surrounding structures including the joint, neurovascular structures, and skin. The extent of the compartmental involvement can be evaluated with MRI to facilitate complete excision of the lesion as well as to demonstrate any skip lesions (i.e., an ipsilateral metastasis within the tumor-bearing bone) and to guide the resection level during surgery. The MRI scan should include the entire bone and the neighboring joints (above and below). Diffusion-weighted imaging sequences are particularly useful in metastatic disease, with their high sensitivity and specificity for detecting cellularity and marrow replacement [39]. This has proven to be useful in the detection of tumor response to therapy and in monitoring bone marrow recovery [40], especially when combined with whole-body MRI. Multiparametric MRI (mpMRI) can be briefly summarized as a method of trying to obtain an ideal three-dimensional (3D) image by combining both anatomical information provided by T1- and T2-weighted, DWI, Dixon-type imaging, with functional information provided by dynamic contrast-enhanced imaging and possibly MR spectroscopic imaging to better assess soft tissue and bony lesions as well as the primary lesion ([41–43]; Fig. 2; Table 2).

**Table 2:** Bone metastasis and the role of imaging (adapted from [44])

Primary tumor	Probability of bone metastases	Action on the bone and bony morphology	Parameter for imaging assessment and functional imaging applications			
			Bone metabolism	Marrow involvement	Diffusion	Glucose metabolism
*Men*						
Prostate	Very high (>50%)	Osteoblastic	+	+	–	Mixed
Lung	High (30–50%)	SCLC: osteoblastic NSCLC: osteolytic	–	+	+	+
Bowel	Moderate (10–30%)	Osteolytic	–	+	+	+
Bladder	High (30–50%)	Variable	+	+	+	–
*Women*						
Breast	Very high (>50%)	Mixed	+	+	+	+
Bowel	Moderate (10–30%)	Osteolytic	–	+	+	+
Lung	High (30–50%)	SCLC: osteoblastic NSCLC: osteolytic	–	+	+	–
Uterus/cervix/ovary	Low	Osteoblastic	–	+	–	–
Melanoma	Moderate (10–30%)	Osteolytic	–	+	+	+

*NSCLC* non-small cell lung cancer, *SCLC* small cell lung cancer

Advanced MRI techniques are probably best performed at dedicated oncology centers equipped with the scanners to perform such studies and the radiological expertise to interpret the findings.

MRI signal characteristics of most MBD [45] include:

- On T1-weighted MRI sequences, low signal within the lesion is more sensitive than osteolysis on CT, as bone marrow infiltration with replacement of fatty marrow precedes bony destruction.
- Osteolytic metastases: high signal on T2-weighted sequences. This can also be seen in necrotic lesions owing to underlying cystic changes.
- Osteoblastic metastases: low or isointense signal on T2-weighted sequences.
- If the lesion has foci of hemorrhage or the primary tumor has high iron content (e.g., in melanoma metastasis), the lesion can demonstrate foci of high signal on T1-weighted imaging. Gradient echo or susceptibility-weighted imaging sequences could be performed to assess this with greater sensitivity**.**
- Often, MBD is associated with increased cellularity and therefore demonstrates restricted diffusion on DWI [39] and varied but increased contrast enhancement following gadolinium administration [46].

## Role of whole-body MRI and DWI in assessment of treatment response

**Fig. 3:**
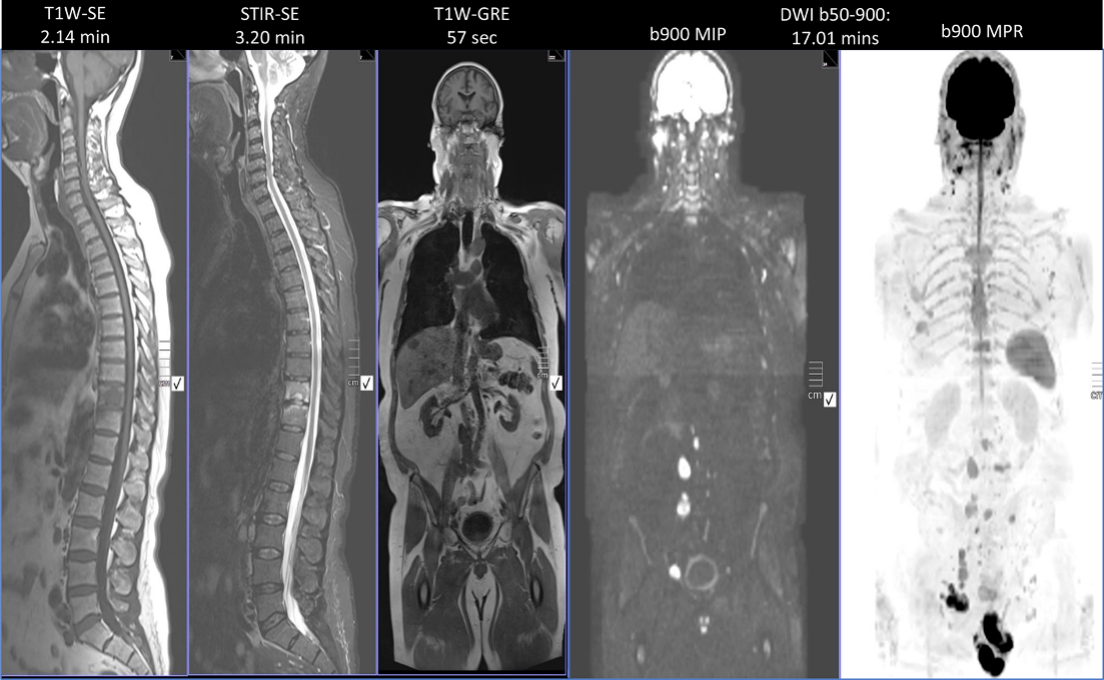
Standard protocols for whole-body magnetic resonance imaging (MRI) for bone metastasis include a short MRI protocol in <30 min (detection) and a comprehensive MRI protocol in 45 min (response). (Courtesy of Prof. Anwar Padhani, © Prof. Padhani. This content is not part of the Open Access License.)

**Fig. 4:**
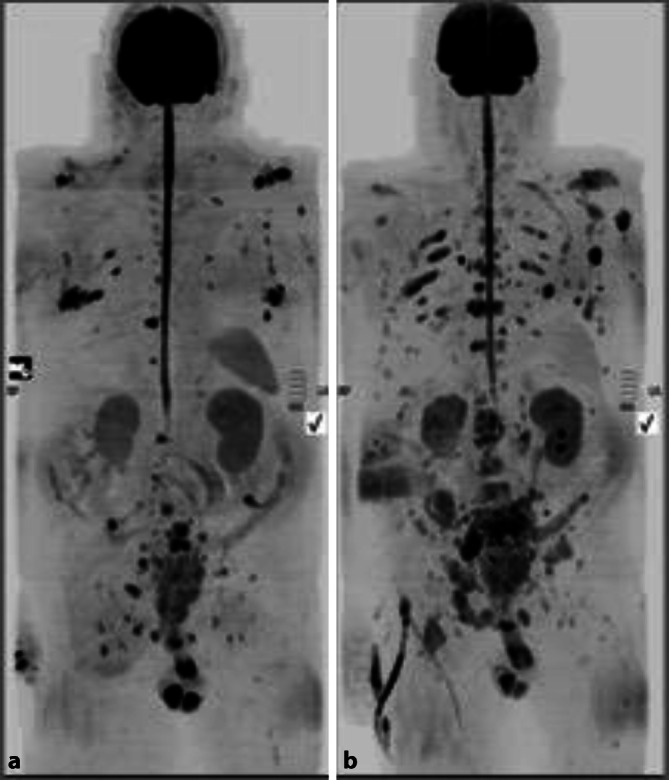
Assessment of disease progression on whole-body magnetic resonance imaging in a 67-year-old male patient with metastatic castrate-resistant prostate cancer on Zoladex and bicalutamide (MAB/CAB). Image **a**: April 2016, Image **b**: August 2016 demonstrating multifocal disease progression with increase in the number and the size of the lesions on diffusion-weighted imaging, with nodal, extra-nodal, and metastatic bone disease as well as further locoregional invasion with bladder and pelvic soft tissue invasion. (Courtesy of Prof. Anwar Padhani, © Prof. Padhani. This content is not part of the Open Access License.)

**Fig. 5:**
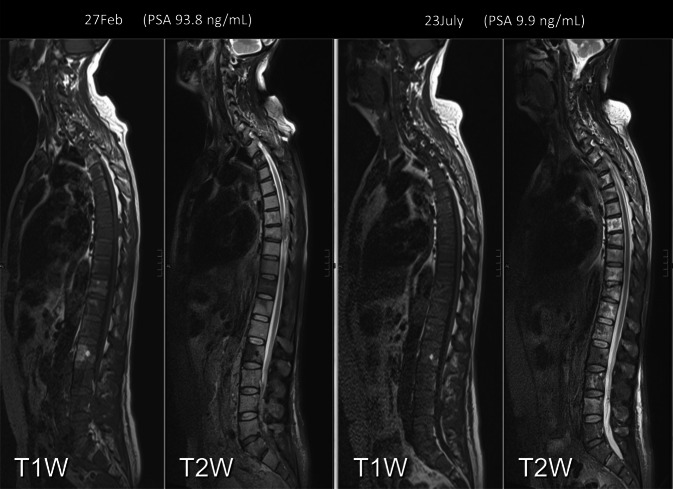
Before and after four cycles of docetaxel, goserelin, and prednisone therapy. Assessment of treatment response in bone and nodes with correlation with prostate-specific antigen (PSA) levels demonstrating partial disease response (important to note when compared with the drastic reduction in PSA ng/ml). (Courtesy of Prof. Anwar Padhani, © Prof. Padhani. This content is not part of the Open Access License.)

**Fig. 6:**
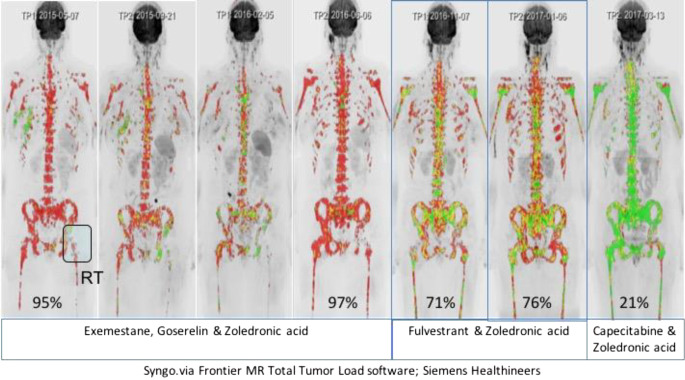
Precision oncology applications of quantitative whole-body magnetic resonance imaging (WB-MRI). A 50-year-old woman with metastatic invasive breast cancer, ER-positive and HER‑2 neu-negative disease was initially treated with first-line hormonal therapy (exemestane, goserelin) and zoledronic acid (exam 2–4). She was switched to second-line hormonal therapy with fulvestrant and zoledronic acid on bone disease progression (exam 5, 6), with response in her bone disease only shown on quantitative WB-MRI. Unfortunately, she also developed liver and pancreas metastases (exam 6) needing therapy change to chemotherapy. Left hip radiotherapy (*RT*) for symptomatic disease was performed. WB-DWI sequences using b‑values of b50, b600, and 900 s/mm^2^ were undertaken to monitor response to treatment. Whole-body tumor load segmentations were undertaken on Syngo.via Frontier MR Total Tumour Load software (Siemens Healthineers) work-in-progress software. The whole-body b900 images are segmented using computed high b‑value images of 1000–1200 s/mm^2^. Extraneous signals (such as the brain, kidneys, and bowel) are removed to leave only recognizable bone disease sites including the right breast and axilla. The color b900 MIP images are overlaid with ADC value classes using the following thresholds: The *green voxels* are values ≥1500 µm^2^/s (representing voxels that are “highly likely” to be responding). The *yellow voxels* are set to lie between the 95th centile ADC value of the pre-treatment histograms (1256 µm^2^/s) and 1500 µm^2^/s, thus representing regions “likely” to be responding. *Red voxels* represent mostly areas that are untreated disease or have no detected response. Hormonal therapies result in brief responses (spotty yellow and green colors), whereas chemotherapy results in marked uniform and widespread increases in ADC values (>1500 µm^2^/s; uniform green colors) observable on exam 7. The *numbers* in each figure represent % *red* (untreated) *voxels* at each time point. (Courtesy of Prof. Anwar Padhani, © Prof. Padhani. This content is not part of the Open Access License.)

Our understanding of molecular processes driving cancer metastasis to the bone is improving [47]. A variety of biological factors resulting in binding of circulating tumor cells to the bone marrow epithelium are thought to be responsible. At a cellular level, once the marrow has been colonized by tumor cells, cytokine-driven interactions between mesenchymal cells and tumor cells alter the normal balanced homeostasis resulting in bone formation (osteoblastic activity) and resorption (osteoclastic activity), thus co-opted to osteolysis and osteosclerosis, resulting in varying imaging phenotypes [41]. Current technologies underserve patients with MBD. Even in 2019, the evaluation of metastatic disease worldwide remains dependent on technologies from the 1970s (namely, bone scans). Although still used to estimate disease burden, bone scans and CT scans employ conventional response criteria and thus tend to underestimate the volume of disease, preventing or delaying changes in ineffective drug therapies, which is expensive and time consuming. This has direct and serious cost implications from a societal perspective. Noninvasive biomarkers to assess therapeutic effects on bone marrow in metastatic disease are therefore urgently required to help guide therapy decisions in primary nonresponders and secondary therapy failure. The use of morphological and size criteria for therapy response in MBD using the aforementioned methods is limited; however, whole-body MRI (WB-MRI) with DWI is promising. The former evaluates bone and soft tissue disease, reflecting biologically important properties such as cellularity. In addition, WB-MRI is widely available and radiation free. It allows not only for metastatic disease detection but also for assessment of therapy efficacy, particularly in cases where CT and bone scans demonstrate little identifiable evidence of disease progression (normal/unchanged bone scan/CT appearances; Figs. 3 and 4). Thus, WB-MRI including DWI is valuable when assessing for bone disease response, facilitating quantification through the use of apparent diffusion coefficient values (ADC, unit µm^2^/s) allowing for objective assessment of therapy response with each drug, chemotherapy cycle, radiotherapy, or ablation technique used, and thereby allowing for more timely modifications in oncological therapy, potential patient outcomes, and health-care costs. It is important to note, however, that interpretation of ADC values is complex, depending on the nature of the tumor and its heterogeneity, particularly in patients with longstanding, relapsing, and remitting disease (Figs. 5 and 6; [41, 48, 49]). Whole-body MRI has the potential to replace current, indirect, ineffective, and wasteful methods of oncological disease assessment in the bone marrow. It could help deliver the promise of precision oncology for patients with malignant bone disease.

## Tc99m bone scan

Radionuclide bone scan detects metabolic bone activity and, in particular, osteoblastic (bone deposition) response. The ability to perform whole-body imaging for the same radiation dose allows us to detect polyostotic disease. The extent of osteoblastic activity can give an indication of disease activity. In addition, intense symmetric uptake in the bones with diminished renal and soft tissue activity (also known as a “superscan”) is an indication of extensive metastatic disease [50].

The sensitivity of the bone scan for lytic lesions depends on the magnitude of the osteoblastic reaction. Pitfalls include post chemotherapy lytic lesions (particularly in breast and lung cancer), bone infarcts and mucinous cystic lesions [51]. Relatively low osteoblastic activity and high osteoclastic activity limit the detection of lesions on bone scans [52].

## Whole-body imaging

### Whole-body MRI, PET CT/WB-MRI/PET MRI FDG- or NaF-PET/CT, and PET/MRI

Multiplanar multimodality whole-body imaging utilizes existing applications of MRI and CT, combined with nuclear medicine studies to provide an overview of MBD load and to ultimately prioritize treatment strategies. Current research focuses on the evolving role of multiparametric imaging in cancer diagnosis, response assessment, and management [53]. Whole-body MRI imaging has increased in popularity thanks to its capacity to detect distant metastases, especially in the axial skeleton, as well as to characterize the lesions, assess their effect on surrounding soft tissues (direct invasion) and study pathological fractures and radiotherapy complications. New technologies with validated novel sequences and improved postprocessing software allow for the quantification of tumor response, and thus enable us to deliver and modulate patient-tailored therapy ([41, 49, 53, 54]; Figs. 3, 4, 5 and 6).

As with other whole-body imaging modalities, such investigations can uncover incidental lesions (so called incidentalomas) that have no relevance to the patient’s clinical condition but may require further investigation. Multiplanar analysis of the lesion increases our sensitivity and specificity in lesion characterization and improves the diagnostic quality of the scan. The role of F‑fluorodeoxyglucose(FDG)- or sodium fluoride(NaF)-PET/CT and PET/MRI in the *initial *diagnostic work-up of bone tumors is still not established. There is an overlap in the maximum standard uptake value (SUV) between benign and malignant tumors. A number of inflammatory lesions can also result in an abnormally high SUV [55]. Sodium fluoride-PET/CT may show a larger number of incidental lesions even when compared with FDG-PET/CT owing to the nature of bone metabolism. Therefore, PET/CT and PET/MR do not yet have a role in the initial differentiation of benign from malignant bone tumors but may aid in problem-solving in cases of suspected local or distant recurrence [50]**.**

## Image-guided biopsy

The multidisciplinary team should include multiple clinicians specializing in the management of skeletal oncology, including radiologists, pathologists, oncological surgeons, and oncologists. Bone biopsies in non-solitary MBD are less commonly performed compared with primary lesions. This is due to the nature of the disease and the pathway of diagnosis. Lesions for which therapy is dependent on confirmation of histology results should be biopsied [40, 53]. Multiple lesions with markedly different radiographic characteristics may require biopsy prior to confirmation of poly-metastasis as a unifying diagnosis. Radiological evaluation of *each lesion* is therefore warranted to enable accurate diagnosis and management and confirm whether or not multiple biopsies are warranted.

Diagnosis with bone biopsy should always be performed after all initial imaging assessments have been completed including MRI and in collaboration with a multidisciplinary team including discussions between histopathologists, radiologists, oncologists, and surgeons to confirm the trajectory of biopsy across anatomical planes. This avoids iatrogenic disease spread and damage of the surrounding structures. The orthopedic oncologist, who performs the definitive surgery, should always be contacted prior to biopsy by the radiologist performing the biopsy. Biopsies should always be performed after complete locoregional imaging, particularly MRI, to ensure preservation of anatomical compartments and reduce the likelihood of iatrogenic spread of locoregional disease. The indications for further histological verification include [56]:

- Cases of more than one primary tumor
- When a primary tumor is not verified (CUP syndrome)
- Primary bone tumor, mono- or oligo-metastases
- Targeted/precision therapies: identification of new targets for treatment, optimizing treatment, identifying and managing tumor recurrence, as well as prediction of tumor response and recurrence rate

Most MBD lesions are imaged and identified after making a histological diagnosis from a confirmed primary lesion. Additional imaging could be performed according to the nature of the primary neoplasm, the treatment plan, and the estimated survival time. A bone biopsy could involve fine-needle aspiration, core-needle biopsy, or incisional biopsy. There is still controversy regarding the diagnostic yield of these biopsy techniques [57]. The current literature has not elucidated a unified optimal biopsy technique for the diagnosis of bone and soft-tissue tumors. However, core-needle biopsy is usually preferable to incisional biopsy because of the low risk of contamination, low risk of complications, and low cost for the procedure. The complication rates reported range between 0 and 10% with an advocated threshold of 2% [57]. In addition, the use of imaging guidance increases the diagnostic accuracy of musculoskeletal biopsies. Computed tomography-guided core-needle biopsy is a safe, accurate, and highly effective procedure for MBD that obviates the need for open surgical biopsy in a significant number of cases. If the result of a percutaneous biopsy is non-diagnostic after a second attempt, an incisional (surgical) biopsy ought to be performed. When combined with functional/fusion imaging, CT guidance is an accurate method of targeting specific regions of interest ([58]; Table 3).

**Table 3:** Biomarkers of bone metastasis (adapted from [44])

	Bone morphology	Bone metabolism	Marrow involvement	Diffusion	Glucose Metabolism
X‑rays	X	–	–	–	–
CT	X	–	–	–	–
SPECT-CT	–	X	X	–	–
MRI	–	–	X	X	–
PET-CT	X	X	–	–	X
PET-MRI	–	X	X	X	X

*CT* computed tomography, *MRI* magnetic resonance imaging, *PET* positron emission tomography, *SPECT* single-photon emission computed tomography

We place a special focus on the commonest sites of MBD—the spine and pelvis—because of the direct relationship of metastasis in these locations with survival, performance status, and mobility. These sites also have a direct impact on the therapy costs for patients affected by MBD in the spine or pelvis when compared with patients with MBD elsewhere.

## Assessing the risk of impending fracture

### SINS score for spine, Mirels’ score for long bones

In 1989, Hilton Mirel proposed a rating system to classify pathologic fracture risk in the axial skeleton [59]. The scoring system is based on four characteristics:

1. Site of lesion
2. Nature of lesion
3. Size of lesion
4. Pain

All the features were assigned progressive scores ranging from 1 to 3 (Tables 4 and 5). This was the first classification that combined the radiological findings, the symptoms, and the impact on the underlying bones (lytic/sclerotic/mixed), backed by statistical evidence to explain the rationale behind the classification.

**Table 4:** Mirels’ score: the classification (adapted from [59])

Variable	Score		
	1	2	3
Location	Upper extremity	Lower extremity	Intertrochanteric
Radiographic appearances	Blastic	Mixed	Lytic
Size (cortical thickness)	<1/3	1/3–2/3	>2/3
Pain	Mild	Moderate	Functional pain (aggravated by movement)

**Table 5:** Mirels’ score: considerations for intervention

Total score	Fracture risk (%)	Recommendations
</=7	<5	Observation and radiotherapy
8	15	Clinically correlate
>/=9	33–100	Prophylactic fixation recommended

According to Mirels, prophylactic fixation is highly recommended for a lesion with an overall score of 9 or greater. A lesion with an overall score of 7 or less can be managed by using radiotherapy and drugs. An overall score of 8 presents a clinical dilemma. The probability of fracture is only 15% and Mirels recommended the attending physician use clinical judgment in such cases and perhaps consider prophylactic fixation on a case-by-case basis.

## Pelvic metastasis

**Fig. 7:**
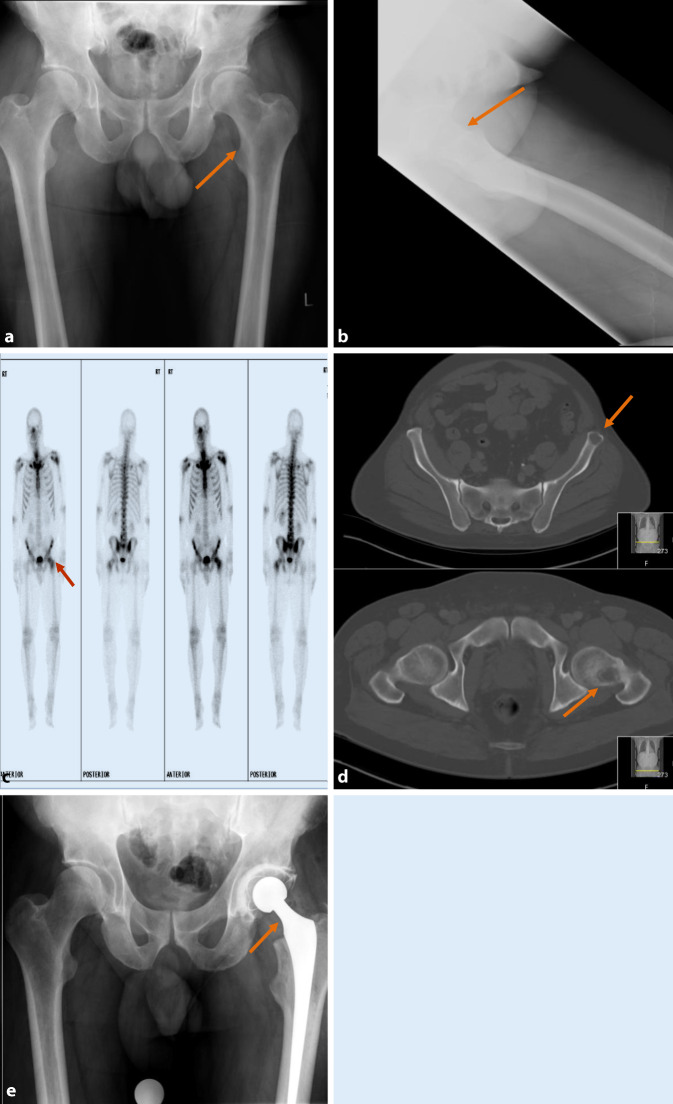
**a,** **b** Plain radiographs, **c** bone scan, **d** computed tomography (CT) scan bone windows, and **e** postoperative radiograph: A patient with a Mirels’ score of 6 for a lucent lesion in the left femur, confirmed on bone scan. The CT scan estimates the lesion to be larger than was seen on plain films and estimated at 2/3rd, raising Mirels’ score to 12. After a clinical consultation, it was established that the patient’s symptoms were constant, not just related to activity. The primary neoplasm was lung with a favorable prognosis. The patient was offered prophylactic total hip replacement with excellent postoperative recovery and remains in remission following cancer therapy

The femur is the most common site of pelvic metastasis with a per-trochanteric lesion in almost 70% of patients. Referral to the orthopedic team is advised for prompt evaluation of the risk of fracture and further advice regarding prophylactic fixation if required (Fig. 7). Mirels’ classification was subsequently validated by Damron et al., who concluded that Mirels’ system is reproducible, valid, and more sensitive than clinical judgment [55].

## Spine metastasis

Andreula and Murrone [25] reported that bone metastases occur in 50% of patients with cancer, and among these, 40–70% are vertebral lesions. In 10% of patients, the neoplastic origin is unknown. In adults, the primary tumors causing vertebral involvement are in the breast (22%), lung (15%), prostate (10%), lymphoma (10%), sarcoma (9%), kidney (7%), and gastrointestinal tract (5%). Most of the metastases are located in the thoracic spine, less frequently in the lumbar, and rarely in the cervical spine (factors 4:2:1). Magnetic resonance imaging is particularly suitable for distinguishing osteoporotic from metastatic spinal fractures with a high degree of diagnostic certainty; however, CT is the modality of choice for stability assessment.

**Fig. 8:**
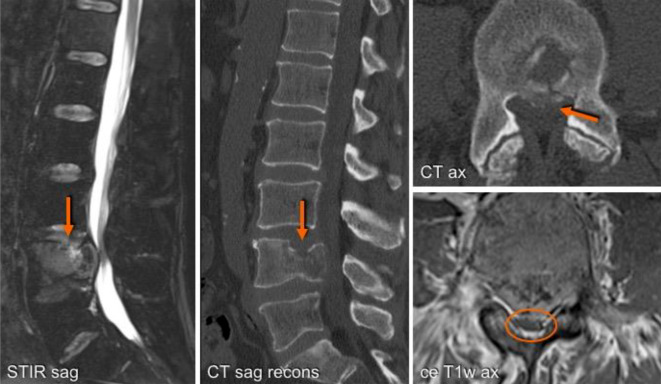
An L4 vertebral lesion replacing fatty marrow mass with an intraspinal portion but no pedicle infiltration. On the sagittal T2-weighted WI-STIR (short-TI inversion recovery weighted imaging) sequence in this 63-year-old patient with metastatic urothelial carcinoma both the hyperintense signal infiltration and, when compared with the CT scan, the pathological fracture are more conspicuous. Axial reconstruction (*CT*) reveals an intraspinal bone fragment with compression of the dural sac, which is best delineated on the contrast-enhanced axial T1-weighted sequence (*ce T1w ax*)

The Spine Instability Neoplastic Score (SINS) is very useful for assessment of spinal instability and helps to decide whether a surgical consultation is necessary, of course after having considered the general condition of the patient, the tumor histology, overall prognosis, and the patient’s preference (Fig. 8; Table 6; [60]). Four major and relatively recent innovations have fundamentally changed the management repertoire for metastatic spine tumors [61]:

1. Advances and integration of spine stereotactic radiosurgery (SSRS) has dramatically improved the locoregional control rate—irrespective of tumor histology and size.
2. Minimally invasive surgical techniques including separation surgery, minimal access techniques, and percutaneous pedicle screw instrumentation and cement augmentation have shortened recovery periods and provided an earlier return to systemic treatment.
3. Spinal instability is defined and validated via the SINS criteria and acknowledged as an independent surgical indication as previously described.
4. Precision oncology: Targeted therapies, such as biologics and checkpoint inhibitors, have significantly improved overall and progression-free survival for most solid tumors and hematologic malignancies.

**Table 6:** SINS Score: Assessment of spinal instability (adapted from [62])

SINS component scoring	Parameter	Score
*Localization*	Junctional zone (occiput-C2, C7-T2, T11-L1, L5-S1)	3
	Mobile spine (C3-C6, L2-L4)	2
	Semi-rigid (T3-T10)	1
	Rigid (S2-S5)	0
*Pain relief with recumbency and/or pain with movement/loading of the spine*	Yes	3
	No (occasional pain but not mechanically provoked)	1
	Pain-free lesion	0
*Bone lesion*	Lytic	2
	Mixed (lytic/osteoblastic)	1
	Osteoblastic	0
	Lytic	2
*Radiographic spinal alignment*	Subluxation/translation present	4
	De novo deformity (kyphosis/scoliosis)	2
	Normal alignment	0
*Vertebral body collapse*	>50% collapse	3
	<50% collapse	2
	No collapse, but >50% of body involved	1
	None of the above	0
	>50% collapse	3
*Posterolateral involvement of the spinal elements*	(Facet, pedicle, or CV joint fracture or replacement with tumor)	–
	Bilateral	3
	Unilateral	1
	None of the above	0
	(Facet, pedicle, or CV joint fracture or replacement with tumor)	–

*SINS* spine instability neoplastic score, *CV* cervical vertebra

**Table 7:** Spine Instability Neoplastic Score (SINS, 0–18)

Total 7 (SINS 0–18)	
Stable situation	0–6
Indeterminate (possibly impending) instability	7–12
Unstable situation	13–18

Semi-rigid segments are nonjunctional segments in the thoracic region that articulate with the rib cage. There is some evidence that surgical decompression for spinal cord compression from metastatic disease before radiation therapy results in improved neurologic outcomes and fewer wound complications. If a patient has neurologic deficit or high-grade spinal cord compression without deficit, surgery is certainly indicated regardless of the SINS (Table 7; [61]).

Spinal alignment describes spinal alignment between motion segments that are affected by tumor. Evaluation of de novo deformity such as kyphosis and/or scoliosis requires knowledge of prior imaging or may be assessed using upright compared with supine radiographs. Bilateral involvement is scored as greater than double the contribution of unilateral involvement because of the destabilizing nature of its effects. Spine stability is only one of many components used to determine management of the patient with a metastatic spine lesion and is perhaps the most difficult component to judge, especially for the non-spinal surgeon clinicians. While plain radiographs may also be helpful, the sensitivity of CT for assessing bony characteristics is much greater and should therefore be utilized whenever possible. Each lesion or general region of neoplastic pathology should be considered in the work-up of spinal neoplastic disease with scores assigned separately to each.

Specific radiological features have been described in an attempt to differentiate metastatic and osteoporotic fractures ([61, 63]; Table 8):

- Convex posterior border of vertebral body indicates malignancy.
- 80–94% specificity.
- Other spinal metastasis → malignancy more likely.
- Concave posterior border → osteoporotic fracture more likely.
- The evidence of a rounded metastatic focus within an adjacent non-collapsed vertebra with similar characteristics of signal intensity increases the likelihood of metastatic pathogenesis.
- Osteoporotic fractures demonstrate the “fluid sign” and tend to be linear or triangular, demonstrating a heavy hyperintense signal on T2-weighted imaging. They are often caused by acute fracture and are therefore often painful at presentation.
- In metastases, the normal fatty marrow of the vertebral body is replaced by diseased tissue. A sharp border to normal marrow on T1-weighted imagine also suggests malignancy.
- Spinal metastases are associated with focal soft tissue masses:
  - Focal, epidural, or paraspinal thickness >10 mm → specificity 93% for malignant fracture.
  - Destruction of pedicles is very specific (sensitivity 80%/specificity 94%).

**Table 8:** Differentiating between osteoporotic and metastatic vertebral body fractures

Etiology	Osteoporotic vertebral body fracture	Metastatic vertebral body fracture
*Location*	Location mostly beneath T7	Location above T7 suspicious
*Morphology of the fracture*	Symmetric fracture (anteroposterior projection or coronal imaging plane) Retropulsion of a posterior bone fragment	Asymmetric fracture (anteroposterior projection or coronal imaging plane) Convex posterior border of the vertebral body pedicle or posterior element involvement evidence of epidural or paraspinal mass and other spinal metastases
*Underlying bony matrix*	Homogeneous bone structure (X-rays, CT) Spared normal bone marrow signal intensity of the vertebral body	Inhomogeneous bone structure (X-rays, CT) → osteolytic or -sclerotic areas
*Background bone marrow*	Remains of normal fatty marrow (T1-weighted images), linear edema along end plate	Replacement of normal bone marrow within the entire vertebral body (T1-weihted images ↓)
*MRI contrast administration*	MR isointensity after contrast application	Marked contrast-enhancement
*Location*	No affection of pedicles or lamina	Affection of pedicles or lamina
*Morphological appearances of the vertebra*	Vacuum (CT) or fluid sign (MRI) in vertebral body Low signal intensity band on T1- and T2-weighted images “fluid sign” or intravertebral “vacuum cleft sign”	Tumor permeation of vertebral border, focal paravertebral growth

In acute vertebral fractures with extensive edema in the bone marrow, the addition of DWI and gradient echo sequences may be beneficial (Figs. 3, 4, 5 and 6).

## Common differential diagnoses for MBD

- In the spine: traumatic and osteoporotic vertebral fractures: MRI is the most helpful radiological investigation in providing the basis for the distinction between metastatic and acute osteoporotic fractures.
- Elsewhere: Primary aggressive and non-aggressive bone tumors.
- Osseous infection: Can occur anywhere and mimic a bone tumor, owing to associated bony destruction and infiltration into the surrounding soft tissues. In spine metastasis, there is often preservation of the end plates and intervertebral discs, which helps to differentiate MBD from infection.

## Clinical management of MBD and imaging strategies

### A “choose wisely” approach to imaging

The American Society of Clinical Oncology (2012/2013) proposed the top five recommendations to the “choose wisely” approach to imaging strategies, which include [64]:

1. Avoid unnecessary anticancer therapy, including chemotherapy, in patients with advanced solid-tumor cancers who are unlikely to benefit, and instead focus on symptom relief and palliative care.
  Data have shown that as many as 10–15% of patients with cancer receive chemotherapy in the last 2 weeks of life. Such care may also postpone patients’ access to palliative care, including hospice care. The ASCO recommends that cancer-directed therapy not be used for patients with solid tumors with the following characteristics: low performance status (3 or 4), no benefit from prior evidence-based interventions, not eligible for a clinical trial, and no strong evidence supporting the clinical value of further anti-cancer treatment. Because further treatment is unlikely to be effective in these patients, emphasis should be placed on palliative and supportive care, which can increase quality of life and, in some cases, extend survival. There is therefore a need to explore further the role of minimally invasive and noninvasive image-guided therapies, including targeted MR-guided high-intensity focused ultrasound (MRg—HIFU), microwave/radiofrequency ablation, and cryotherapy. Further randomized controlled studies are warranted to assess which of these new evolving therapies is most beneficial for each cohort of patients.
2. Do not perform PET, CT, and radionuclide bone scans in the staging of early prostate cancer at low risk for metastasis.
3. Do not perform PET, CT, and radionuclide bone scans in the staging of early breast cancer at low risk for metastasis. Instead, whole-body MRI is indicated to assess and monitor disease response and possible relapse, without subjecting the patient to high radiation doses.
4. Do not perform surveillance testing (biomarkers) or imaging (PET, CT, and radionuclide bone scans) for asymptomatic individuals who have been treated for breast cancer with curative intent. The ASCO authors note that false-positive results are very common with these tests and can lead to invasive procedures, over-treatment, and misdiagnosis that can severely affect quality of life.
5. Avoid administering white blood cell stimulating factors to patients who have a very low risk for febrile neutropenia (less than 20%).

However, in 2019, these recommendations were still not in daily use (e.g., Simos 2014: 1/3 of patients with early breast cancer underwent a bone scan, 1/3 chest X‑rays and abdominal ultrasonography, and 1/3 CT of the chest, abdomen, and pelvis). Data suggest white blood cell stimulating factors are often not used according to evidence-based guidance, costing health systems millions and potentially causing unnecessary side effects for patients (e.g., bone aches, low-grade fever, and malaise). In one study, 10% of patients at low risk (less than 20%) for febrile neutropenia received these treatments. Another study showed that Medicare spent at least $ 40 million in 2005 on CSF therapy for women with ER-positive breast cancer, even though studies have not demonstrated a benefit for such patients. In addition to the aforementioned points, identifying patients with extensive locoregional disease as a separate subgroup facilitates management and referral pathways in those who may benefit more from nonsurgical management of their skeletal disease.

### Surgical options

Surgery can either aim toward being curative (complete excision of the lesions) or for mechanical stabilization (internal fixation and/or cementoplasty) or palliative care.

## Precision oncology

Precision oncology, defined as genetic profiling of tumors throughout the therapeutic journey to identify and target genetic alterations and focal mutations as well as variation in response to therapies, is a widely accepted and rapidly evolving clinical approach to MDB. The goal of precision medicine is to deliver the precise cancer treatment to the correct patient at the precise dose and the exact time [65] and therefore it promises more accurate histological correlation and management [41, 49]. Although seemingly costlier, the precision oncology approach may actually be cost effective by offering (a) earlier and more precise diagnosis and staging, (b) specific therapies to a particular tumor type, and more importantly patient-specific doses (of drug or radiotherapy), (c) earlier detection of associated complications, and (d) detection of drug resistance and tumor response for both therapeutic and counselling purposes [66].

Further research on drugs, mutations, and genetic influences is warranted to assess their mid- to long-term benefits and impact on the course of disease.

## Summary

Bone metastases represent a major health-care issue and their multidisciplinary management needs the participation of orthopedic surgeons, pathologists, oncologists, radiotherapists, and radiologists [26]. The radiologist has a key role in the decision-making process according to the tumor entity, tumor biology, and general condition of the patient by choosing the best individual imaging modality after interdisciplinary discussion (“choosing wisely”) and by offering minimally invasive treatment options, such as for pain control [67]. An early and accurate diagnosis of bone metastases is therefore crucial; however, the pattern of bone metastases is very heterogeneous and necessitates good knowledge of the possibilities and limitations of each imaging modality.

## Practical conclusions

- Bone metastases impose a high social and health-care cost burden.
- Metastatic bone disease is common. It is therefore important for the reporting radiologists to critically evaluate the bones on all imaging examinations performed regardless of the clinical indication.
- Magnetic resonance imaging of the spine should be considered in patients presenting with new focal or widespread neural compromise.
- Scoring systems such as the one by Mirels or the Spine Instability Neoplastic Score help to decide whether conservative or surgical therapy of bone metastases is necessary.
- Currently, whole-body MRI, whole-body PET/MRI and PET/CT have the highest sensitivity and specificity for detecting bone metastases.
- Evolving imaging techniques improve targeted approach to patient care and the evolution of precision oncology.
- Imaging may help guide the oncology team to better select the palliative group in whom more aggressive and invasive therapies have been proven to be ineffective and costly in the last precious weeks/months of life.
- Multidisciplinary meetings and discussions have a significantly positive impact on patient care. The more we know, the better our decisions are based on collective rather than individual clinician experiences.
- Dedicated orthopedic metastasis services are required in every major cancer center to expedite care of patients with acute or impending fracture.
